# A New Case of Syringocystadenocarcinoma Papilliferum: A Rare Pathology for a Wide-Ranging Comprehension

**DOI:** 10.1155/2014/453874

**Published:** 2014-05-15

**Authors:** Beatrice Paradiso, Enzo Bianchini, Pierangelo Cifelli, Luigi Cavazzini, Giovanni Lanza

**Affiliations:** ^1^Department of Morphology, Surgery and Experimental Medicine, Section of Anatomic Pathology, University of Ferrara and S. Anna University Hospital Via Aldo Moro, 8, 44124 Cona, Ferrara, Italy; ^2^Department of Medical Sciences, Section of Pharmacology and Neuroscience Center, University of Ferrara, Via Aldo Moro, 8, 44124 Cona, Italy; ^3^National Institute of Neuroscience, Italy; ^4^Ri.MED Foundation, Palermo, Italy

## Abstract

We report a new case of p63/cytokeratin 7 (CK7) positive syringocystadenocarcinoma papilliferum (SCACP), on the shoulder of an 88-year-old man, with superficial dermal infiltration and squamoid differentiation. We describe the 24th case of SCACP, the malignant counterpart of syringocystadenoma papilliferum (SCAP). At the present, we do not know whether SCACP arises from eccrine or apocrine glands because of the contrasting opinions in the literature. Only few histochemical and ultrastructural studies have previously advised that SCACP could arise from pluripotent stem cells. Through our case, we wish to suggest the stem cell-like properties of the syringocystadenocarcinoma papilliferum. This rare neoplasm shows two different patterns of stem cell marker expression in the glandular and squamous components, respectively. For the double phenotype of SCACP, we propose it like an intriguing model to study histogenesis and stem cell properties for more wide-ranging epithelial tumors.

## 1. Introduction

Syringocystadenocarcinoma papilliferum (SCACP) is a rare malignant form of syringocystadenoma papilliferum (SCAP), cutaneous adnexal neoplasm defined by Stokes in 1917. Some authors are unsure about the malignant transformation in syringocystadenoma papilliferum but, recently, Hoekzema et al. [1] have presented a well-documented SCACP case arising from preexisting SCAP, which appeared to be an epidermal nevus. SCAP mostly occurs in the head and neck region and is associated with a nevus sebaceous (NS) of Jadassohn in 30–40% of cases, but may also appear* de novo* (without NS) on other parts of the body.

There is an increasing evidence for an apocrine histogenesis of SCAP and SCACP [2] but the possibility of an eccrine origin from sweat glands for a few cases seems likely. Another theory suggests the apoeccrine glands to be the origin of these tumors that could be skin hamartomas [1, 3]. Very few histochemical and ultrastructural studies propose that SCACP arises from pluripotent cells [1, 3, 4]. A little map of the different theories is shown in Table 1.

Histologically, the connection to the skin surface, by typical epidermal transition, the decapitation on the luminal surface of inner layer cells, the tumor connection to folliculosebaceous structures, the layering of apocrine glands in the underlying tissues, and positive reactions to diastase-resistance periodic acid-Schiff (PAS) provide evidence of apocrine differentiation of SCACP [1, 2].

Herein, we report a case of SCACP occurring on the left shoulder of an 88-year-old European male patient.

## 2. Materials and Methods

### 2.1. Case

An 88-year-old man presented with a single nodule on the left shoulder. He had had multiple lesions for 20 years of bowenoid actinic keratosis on the face, the forehead, and the left ear. In the last 13 years, the patient underwent prostatectomy for adenocarcinoma, score 8 (5+3) according to Gleason and squamous cell carcinoma on the left eyelid surgically treated. The man stated that the lesion began as a bean-sized papule and had increased gradually in size with time. It also became painful. Physical examination revealed a single, 1.5 × 1.5 cm, erythematous, dome-shaped, and firm tumor surrounded by normal skin on the left shoulder. Regional lymph nodes were not palpable, and the physical examination did not reveal any remarkable findings except for the mass. The patient had no clinical or radiographic evidence of a head and neck or lung primary tumors. He died after two years of old age.

### 2.2. Histology

The present skin tumor was clinically analyzed and then resected tumor specimens were fixed in 10% buffered formalin and embedded in paraffin. Sections of 5 *μ*m were subjected to conventional microscopic histochemical and immunohistochemical observations.

PAS reaction, with and without diastase digestion, was also carried out.

For the immunostaining, we used an automatic and clinically validated instrument based on Ventana Benchmark Ultra systems from Roche Tissue Diagnostics. Immunohistochemistry was performed by means of a new enhanced sensitivity biotin-free multimer technology system, based on direct linkers between peroxidase and secondary antibodies (ultraView Universal DAB Detection Kit, Ventana Medical System). The following primary antibodies were used prediluted: antipancytokeratin (PCT), anticytokeratin 7 (CK7), anticytokeratin 5/6 (MES), anti-e-cadherin (epithelial) (e-cad), antiepithelial membrane antigen (EMA), anticarcinoembryonic antigen (CEA), anti-p63, anti-S100 by Ventana; anti-CD117 (c-kit) (Cell Marque), and anti-Ki-67 antigen (MIB-1) by DAKO.

For immunofluorescence, sections were dewaxed, rehydrated, as described above, and unmasked using a commercially available kit (Unmasker, Diapath). Slices were incubated with Immunological Sciences primary antibodies, mouse monoclonal anti-CD44 1 : 50, and rabbit polyclonal anti-CD133 4 *μ*L/mL and then incubated with Molecular Probes secondary antibodies, 1 : 100, goat anti-mouse, green fluorescent Alexa Fluor 488-conjugated and goat anti-rabbit, and red fluorescent Alexa Fluor 594-conjugated and finally counterstained with 0.0001% DAPI (4′, 6-diamidino-2-phenylindole), in accordance with the preview published protocol [20].

## 3. Results

All the immunoprofiling data about the normal skin, the benign lesion, and the carcinomatous component are summarized in Table 2.

Hematoxylin and eosin staining of histological sections showed squamous cancer cells in the dermis to be gradually deepening. The squamous budding generated internal cavities giving rise to glandular papillary structures. These tumoral squamous-papillary glands were positively stained by anti-PCT antibody in the outer layer of budding; the glandular component was heterogeneously stained with accentuation of the membranes. Anti-CK5/6 strongly stained the transition area from stratified squamous epithelium to neoplasia; the squamous solid component and the glandular structures were moderately stained. The transition area from stratified squamous epithelium to neoplasia was strongly positive for CK7; the glandular budding and the papillary components were intensely stained by CK7 antibody; contemporaneously, the squamous solid neoplasia appeared negative. E-cad staining showed an increase of pericytoplasmic positivity in the transition area from stratified squamous epithelium to neoplasia but completely negative was the infiltrative portion. Neoplastic areas, in contrast with normal epidermis, resulted in totally stained EMA. Syringocystadenocarcinoma was completely negative for CEA and robustly positive with patchy nuclear for p63 staining. By anti-c-kit antibody, we obtained highly positive staining of basal layer of neoplastic budding; the glandular and papillary structures were heterogeneously positive but almost negative and/or speckled positive was the squamous solid component. The evident desmoplastic stromal reaction accompanied the budding and contained numerous plasma cells and lymphocytes. The epithelial transition and the decapitated secretion provided evidence of apocrine differentiation; the cytological atypia gave a precious suggestion of the neoplasm. An invasive squamous component coexisted with glandular elements typical for adenocarcinomas. The dual p63-CK7 immunohistochemical positivity was useful in confirming the diagnosis definitive of syringocystadenocarcinoma papilliferum.

Nestin, CD44, and CD133 antigens, markers of stem cell properties, were overexpressed in the neoplasm and could suggest a tumor origin by pluripotent stem cells.

## 4. Discussion

**Figure 1:**
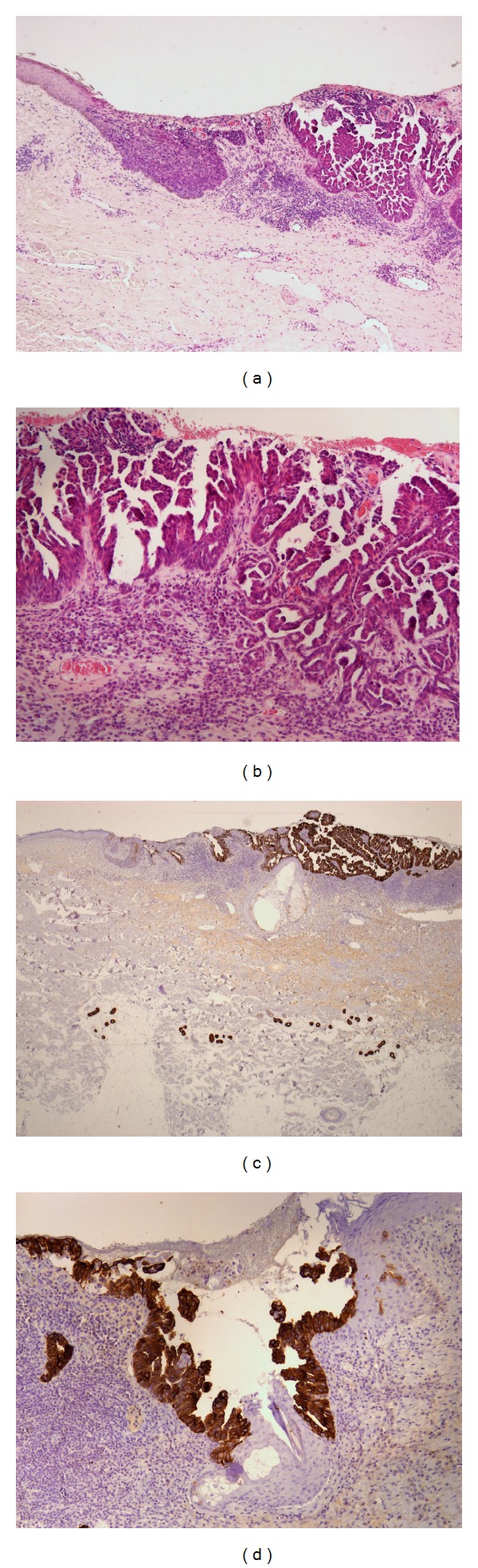
Analysis of a skin syringocystadenocarcinoma papilliferum specimen. (a) Specimen shows squamous cancer cells in the dermis gradually deepening. The squamous budding generates internal cavities giving rise to glandular papillary structures (hematoxylin-eosin [H&E], original magnification ×40). (b) Note the adenopapillary infiltrative structures ([H&E], original magnification ×100). (c) Immunohistochemical staining demonstrates CK7 at the glandular budding forming the papillary components (original magnification ×40). (d) The squamous solid neoplasia persists negative for CK7 (original magnification ×100).

**Figure 2:**
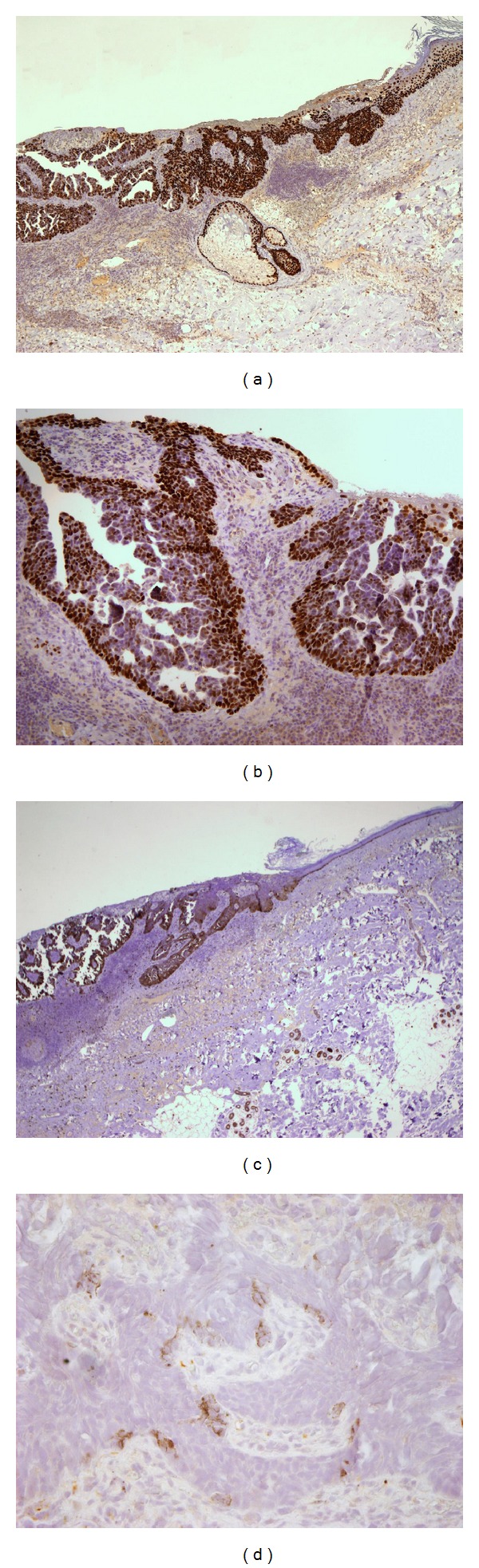
Analysis of a skin syringocystadenocarcinoma papilliferum specimen. (a) p63 antibody stains the transition area from epidermis epithelium to the squamous neoplasm and the basal layer of glandular component (original magnification ×40). (b) The papillary structures are moderately positive and/or negative for p63 (original magnification ×100). (c) Immunohistochemical staining demonstrates c-kit at the basal layer of neoplastic budding; almost negative, only with speckled positive cells, is the squamous solid component (original magnification ×20). (d) A few positive cells of the squamous neoplasia are stained by Nestin antibody, stemness marker (original magnification ×400).

We report a second case of syringocystadenocarcinoma with some unique histologic features; preview described by Leeborg et al. [2] demonstrates the p63 positive-gradual transition area from keratinizing squamous epithelium to neoplasm (Figures 2(a) and 2(b)). Syringocystadenocarcinoma papilliferum is a rare neoplasm of doubtful (apocrine versus eccrine) origin and is the malignant counterpart of the more common benign syringocystadenoma papilliferum. Histologically, syringocystadenocarcinoma papilliferum resembles syringocystadenoma papilliferum and solid areas of tumor may be present, in addition to the glandular, cystic, and papillary areas that deepen like dermal cleft by a squamous transition epithelium (Figures 1(a) and 1(b)). The glandular epithelial cells show malignant cytological features, such as high nuclear to cytoplasmic ratios and nuclear irregularity, and, in particular, they are PAS diastase positive, with decapitated cytoplasm, confirming the apocrine origin. Our case is distinctive in several aspects. It is the second report, to our knowledge, to document the presence of a high-grade cell component originally considered to be a high-grade squamous cell carcinoma and then demonstrated adenocarcinoma [2]. It is the first case to provide immunohistochemical evidence of stem cell features and epithelial-mesenchymal transition- (EMT-) like properties.

To analyze the biological and carcinogenetic property of our tumor case, we have used a series of antibodies fit for recognizing epithelial expression, proliferation index, and stem cell properties.

Like Leeborg et al. suggested [2], we have found CK7 and p63 positive-cells in continuous stream from normal epithelium to neoplastic adenocarcinoma clefts, that is, the squamous-glandular transdifferentiation of the tumor (Figures 1(c), 1(d), 2(a), and 2(b)). Furthermore p63 positivity supports the primary origin of the squamous and/or cutaneous-adnexal tumor and distinguishes it from metastatic adenocarcinoma [21]. Simultaneously, we have demonstrated, for the first time, a possible staminal origin of syringocystadenocarcinoma papilliferum by c-kit, nestin, CD44, and CD133 immunopositivity.

The transmembrane tyrosine kinase receptor c-kit (CD117) is a 145–165-kD proto-oncogene, related to platelet-derived factor receptor and the colony stimulatory factor receptor. The primary ligand for c-kit is stem cell factor (SCF) or also named mast cell growth factor and steel factor. Evidence supports [22] the existence of CD117 as putative marker of mesenchymal stem cells and/or cancer stem cells. Overexpression of c-kit has been demonstrated in several human tumors such as gastrointestinal stromal tumor (GIST), small-cell lung cancer, colorectal cancer, Ewing's tumor, and chronic myelogenous leukemia (CML). Other c-kit positive-normal cells include epithelial cells in skin adnexa, breast, and subsets of cerebellar neurons.

In our case, we observed c-kit positive-epidermal epithelium basal layer connected with neoplastic budding by c-kit positive-tumor cells that presented the same pattern of positivity for CK7 and p63, too (Figures 2(c), 1(c), and 2(a)). The positivity for c-kit was robust and more intense in the basal layer of the glandular neoplastic budding, in continuity with the c-kit positive-normal basal layer of epithelium. We observed heterogeneously intense-moderate amount of staining of glandular and papillary structures; almost negative, with limited occurrence and speckled positivity appeared the squamous solid component. Interestingly, we found some positive spindle cells in perineoplastic stroma (see Figure S1 in Supplementary Material available online at http://dx.doi.org/10.1155/2014/453874). Recently, it has been reported that c-kit plays a critical role in the invasion and metastasis of salivary adenoid cystic carcinoma (ACC) and may participate in EMT of salivary ACC [23]. Furthermore e-cad staining was interestingly expressed (data not shown). We found, as recent literature has proposed [24], a different level of this central component of cell junctions-adhesion molecule: plasmalemmal positivity in the normal tissue and in the transition area from stratified squamous epithelium to neoplastic budding with occasionally negative neoplastic cells; we observed a gradual decreasing of e-cad positivity into neoplasia, with focally negative neoplastic glands. Totally unstained appeared the invasive papillary component. All of these data together either with c-kit pattern or with the spindle cell morphology in the front of infiltration and the surrounding desmoplastic stromal reaction (Figures 2(c), S1, and 1(b)) could be convincing features of EMT-like process [25, 26]. The EMT paradigm is a developmental pathway by which epithelial cells are transdifferentiated to mesenchymal cells during embryogenesis, tissue remodelling, and wound healing. Recently, an interesting hypothesis considers EMT as a more general event that has been implicated in all types of carcinoma and provides them with an additional survival advantage. By means of EMT, the epithelial tumor cells would transdifferentiate into myofibroblasts, losing their malignant phenotype but producing the desmoplastic stroma which is essential for tumor growth, invasion, and metastasis. In human cancer, the phenomenon is rarely demonstrated because, after the infiltrative spreading, it is fast reverted by mesenchymal epithelial transition (MET) program, in cancerous epithelial phenotype [27–29]. It has been observed that, during the EMT process, cancer epithelial cells acquire stem cell-like traits and appear positive for putative cancer stem cell (CSC) markers, resulting in a migratory cell phenotype [30, 31]. So EMT-like properties of carcinoma are more persuasive, when they are associated with an increase of putative stemness marker expression. For this reason, we have analyzed nestin, CD44, and CD133 antigens [21, 24, 32–34].

Nestin, VI intermediate filament protein and marker of precursor cells, is also expressed in the stem cells of the hair follicle and in the endothelial cells; moreover, nestin is expressed in the peritumoral stroma of basal cell carcinomas [21, 33]. In our case, we found in bulge squamous area of neoplasia some nests of nestin-positive stroma cells with mesenchymal phenotype (Figure S2) but also convincing clusters of nestin positive-cancer stem cells in the epithelial neoplasia (Figure 2(d)).

**Figure 3:**
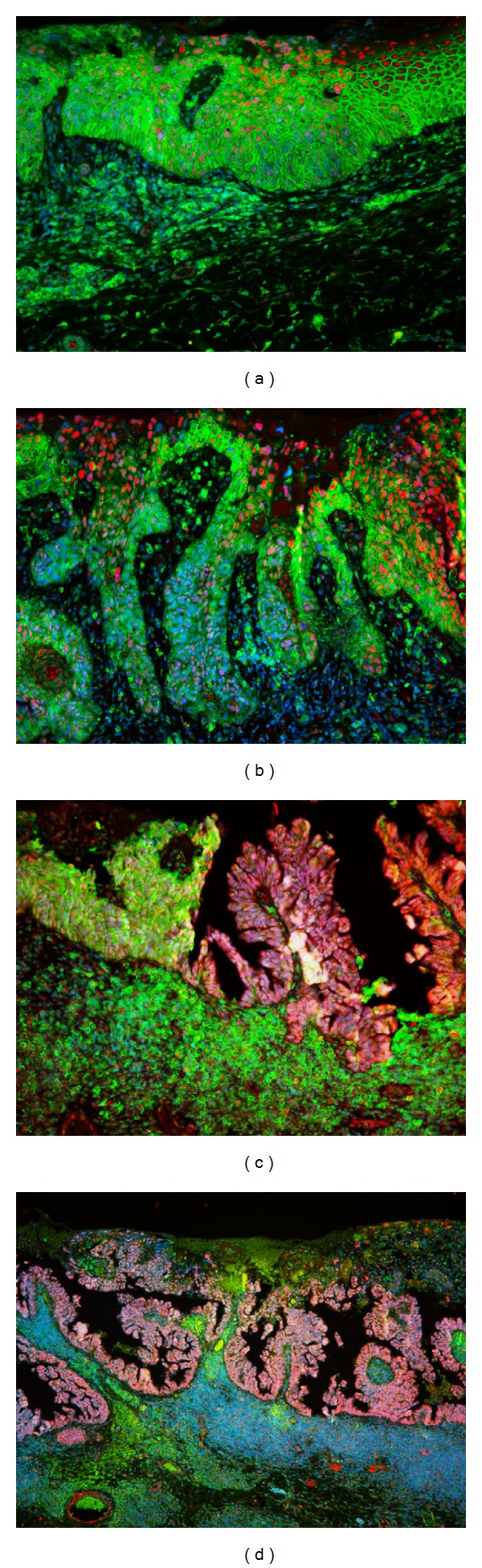
Fluorescence microscopy analysis of the staminal property of syringocystadenocarcinoma papilliferum. Immunofluorescent staining demonstrates anti-CD133 (in red) and anti-CD44 (in green) antibodies; the nuclei are contrasted by DAPI (in blue). (a) The tumoral squamous portion shows membranous CD44 overexpression (original magnification ×200). (b) The transition area presents squamous budding with decreasing of CD44 positivity but increasing of nuclear-cytoplasmic CD133 expression (original magnification ×200). (c) Bifront-like staining showing CD44 more represented in the squamous area and CD133 stronger in the papillary component (original magnification ×200). (d) Overview of CD133 positive-cystic and papillary structures (original magnification ×100).

The CD44 antigen is a cell-surface glycoprotein involved in cell-cell interactions and cell adhesion and migration, for these properties participate in a wide variety of cellular functions including lymphocyte activation, recirculation and homing, hematopoiesis, and tumor metastasis. Recently CD44 has been recognized as a marker of putative cancer stem cells [35], in particular of squamous phenotype; CD44 high expression plays a crucial key role in initiation, malignant transformation, and EMT-like program [24, 32, 36]. An interesting aspect of our case consists of evident overexpression of CD44 in squamous component of neoplasia, with spreading positivity in the perineoplastic stroma cells(Figure S3). Almost completely negative for CD44 appeared the glandular and papillary components (Figure 3).

CD133, prominin 1, is pentaspan 120-kDa transmembrane glycoprotein and considered to be a hematopoietic and ubiquitarian stem cell marker, although its biological function remains largely unknown. However, this protein has been identified as a potential cancer stem cell marker in the brain, colon, prostate, pancreas, and, recently, the skin [37]. Until now, the correlation between CD133 expression and clinicopathological features of tumors, like increased tumorigenic and patient survival, persist undemonstrated. Only few data in the literature describe the clinical significance of the expression patterns of CD133 (membranous and/or cytoplasmic) [38] but it is clear that aberrant overexpression is pathognomonic for neoplasia. In particular, we found as a strong uniform pattern of nuclear-cytoplasmic CD133 overexpression in the adenocomponent that had lost the CD44 membranous positivity during the glandular neoplastic transdifferentiation (Figure 3).

By PCT immunostaining, we demonstrated the epithelial origin of the tumor because PCT immunopositive-cells appeared in normal epithelium and in continuity with the external layer of the neoplasm. This suggests the primary epithelial budding of adenocarcinoma. (data not shown). CD133, prominin, increased in squamous carcinoma but more prominently in cystic-glandular neoplasia. This pattern, associated with specular behavior of CD44, can suggest the development and evolution of glandular part from squamous component.

By intense immunopositivity for CK7, p63, and c-kit and the particular neoplastic stromal reactivity, we hypothesized that this tumor could have EMT-like properties. Furthermore, the p63/CK7 staining has also highlighted the squamous transdifferentiation of this adnexal-like neoplasm. Since the normal basal layer of epithelium was c-kit positive like the neoplastic component, we suggest that this tumor could derive from pluripotent-like cells [39], also with the support of high positivity for nestin, CD133, and CD44. Typical PCT immunostaining demonstrated the ontogenetic behavior of epithelial-adnexal neoplastic budding. In front of these original data, we suggest to reanalyze the syringocystadenocarcinoma papilliferum cases in the light of new evidences regarding histogenesis and stem cell-like properties. Syringocystadenocarcinoma papilliferum is a very infrequent skin tumor but could be a good model to verify the existence of the cancer stem cells and their transdifferentiable properties in skin neoplasms.

## Supplementary Material

EMT represents a general physiopathologic event implicated in all types of carcinoma and provides them an additional survival advantage. By means of EMT the epithelial tumor cells can transdifferentiate into myofibroblasts, producing the desmoplastic stroma which is essential for tumor growth, invasion and metastasis. During the EMT process, cancer epithelial cells acquire stem cell-like traits and appear positive for putative cancer stem cell (CSC) markers, resulting in a migratory cell phenotype. So EMT- like properties of carcinoma, showed by c- kit staining, are more persuasive when they are associated with an increase of putative stemness marker expression. For this reason we have analyzed nestin, CD44 and CD133 antigens.

## Figures and Tables

**Table 1:** Histogenetic theories of syringocystadenocarcinoma papilliferum.

Source, year	Eccrine and/or apocrine histogenesis
Dissanayake and Salm, 1980 [5]	Eccrine: in situ carcinoma

Dissanayake and Salm, 1980 [5]	Eccrine

Seco Navedo et al., 1982 [6]	Apocrine

Numata et al., 1985 [7]	Apocrine

Bondi and Urso, 1996 [8]	Eccrine

Ishida-Yamamoto et al., 2001 [4]	In situ carcinoma, near the anal apocrine glands, small number of tumor cells diastase-resistant, origin from a pluripotential germinative cell

Arai et al., 2003 [9]	In situ carcinoma, diastase-resistant PAS-positive

Chi et al., 2004 [10]	Either pluripotential appendageal cells or primitive apocrine glands

Woestenborghs et al., 2006 [11]	Eccrine: in situ carcinoma

Kazakov et al., 2007 [12]	In situ carcinoma arising in association with SCAP and sebaceous carcinoma

Park et al., 2007 [13]	SCACP originates from apocrine glands

Langner and Ott, 2009 [14]	In situ carcinoma, luminal columnar cells with decapitation secretion

Kazakov et al., 2010 [15]	Apocrine: 5 new cases: in situ carcinoma and invasive tumors; squamous differentiation

Leeborg et al., 2010 [2]	Apocrine: in situ carcinoma and invasive tumor; squamous differentiation

Sroa et al., 2010 [16]	Eccrine

Aydin et al., 2011 [17]	Eccrine

Hoekzema et al., 2011 [1]	Apocrine: from pluripotent cells

Hoguet et al., 2012 [18]	Apocrine

Zhang et al., 2012 [19]	Apocrine with squamous differentiation

Present case, 2014	Apocrine: from pluripotent cells by epithelial mesenchymal transition

**Table 2:** Antigen expression of syringocystadenocarcinoma papilliferum.

Immunohistochemical studies	Normal skin epithelium	Syringocystoadenoma	Syringocystoadenocarcinoma
PCT	Completely positive	Completely positive	Positive outer layer of budding; heterogeneously stained glandular component with accentuation of the membranes

CK5-6	Positive basal and spinous layers	Positive basal layer	Strongly stained transition area from stratified squamous epithelium to neoplasia; heterogeneously and moderately stained squamous solid component and glandular structures

CK7	Negative	Negative	Positive transition area from stratified squamous epitelium to neoplasia; negative squamous solid neoplasia with weak speckled area; positive glandular budding and papillary components

e-cad	Positive epidermidis epithelium; negative keratin layer	Increased pericytoplasmic positivity of the transition area from stratified squamous epitelium to neoplasia with focally negative neoplastic cells	Decreased staining of the neoplastic glands and papillary component and negative infiltrative portion

EMA	Negative	Focally positive	Strongly stained transition area and glandular and papillary components

CEA	Negative	Focally positive	Negative

p63	Almost positive throughout the whole epidermis epithelium, with the strongest expression in basal and suprabasal layers	Positive in discontinuous basal layer	Strongly stained transition area from stratified squamous epithelium to neoplasia; highly positive squamous neoplasm and basal layer of glandular component; moderately positive papillary neoplasia

c-kit	Positive basal layer of epidermis	Positive basal layer of neoplasia	Positive; highly positive basal layer of neoplastic budding; heterogeneously intense-moderate amount of staining of glandular and papillary structures; almost negative, limited occurrence and speckled staining of the squamous-solid component; some positive spindle cells in perineoplastic stroma

S100	Focal positivity of basal layer	Focal positivity	A few positive cells in the squamous component and in perineoplastic stroma

K67	Focal positivity of basal layer	Moderately positive	Completely positive

Nestin	Negative; positive-endothelial cells and hair follicles	Negative; positive-endothelial cells and hair follicles	A few positive cells in the squamous component and in perineoplastic stroma

CD133	Almost negative; very rare positivity in the granular layer epidermis	Moderately positive	Aberrant cytoplasmic overexpression, more intense in the glandular and papillary components

CD44	Transmembrane positivity with the strongest expression in basal layers	Faintly positive	Increasing of pericytoplasmic expression in the neoplastic squamous component; almost unstained glandular component; positivity in the stroma
