# Supplementary material for: A New Case of Syringocystadenocarcinoma Papilliferum: A Rare Pathology for a Wide-Ranging Comprehension
Source: Case Rep Med. 2014 May 15;2014:453874. doi: 10.1155/2014/453874 (PMC4052556; doi:10.1155/2014/453874)

**A new case of Syringocystadenocarcinoma Papilliferum. A rare pathology for a wide-ranging comprehension**

**Supplementary Figure Legend**

**Figure S1.** Immunohistochemical staining demonstrates c-kit at the basal layer of neoplastic budding with diffuse, scattered c-kit positive spindle cells in the perineoplastic stroma (original magnification ×100).

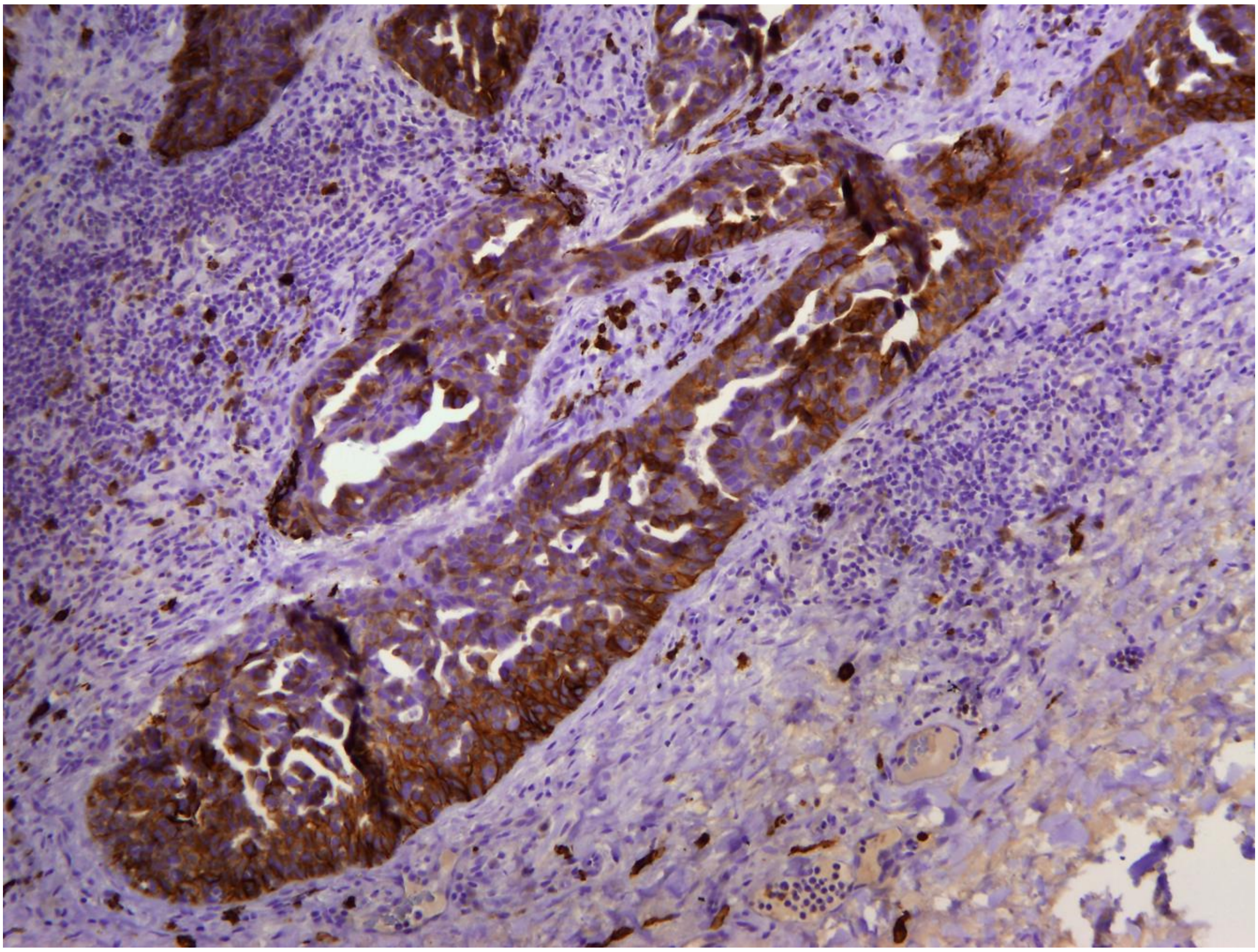

**Figure S2.** Some stroma cells with mesenchymal phenotype and few cell in the bulge squamous area of neoplasia are Nestin- positive (original magnification ×400).

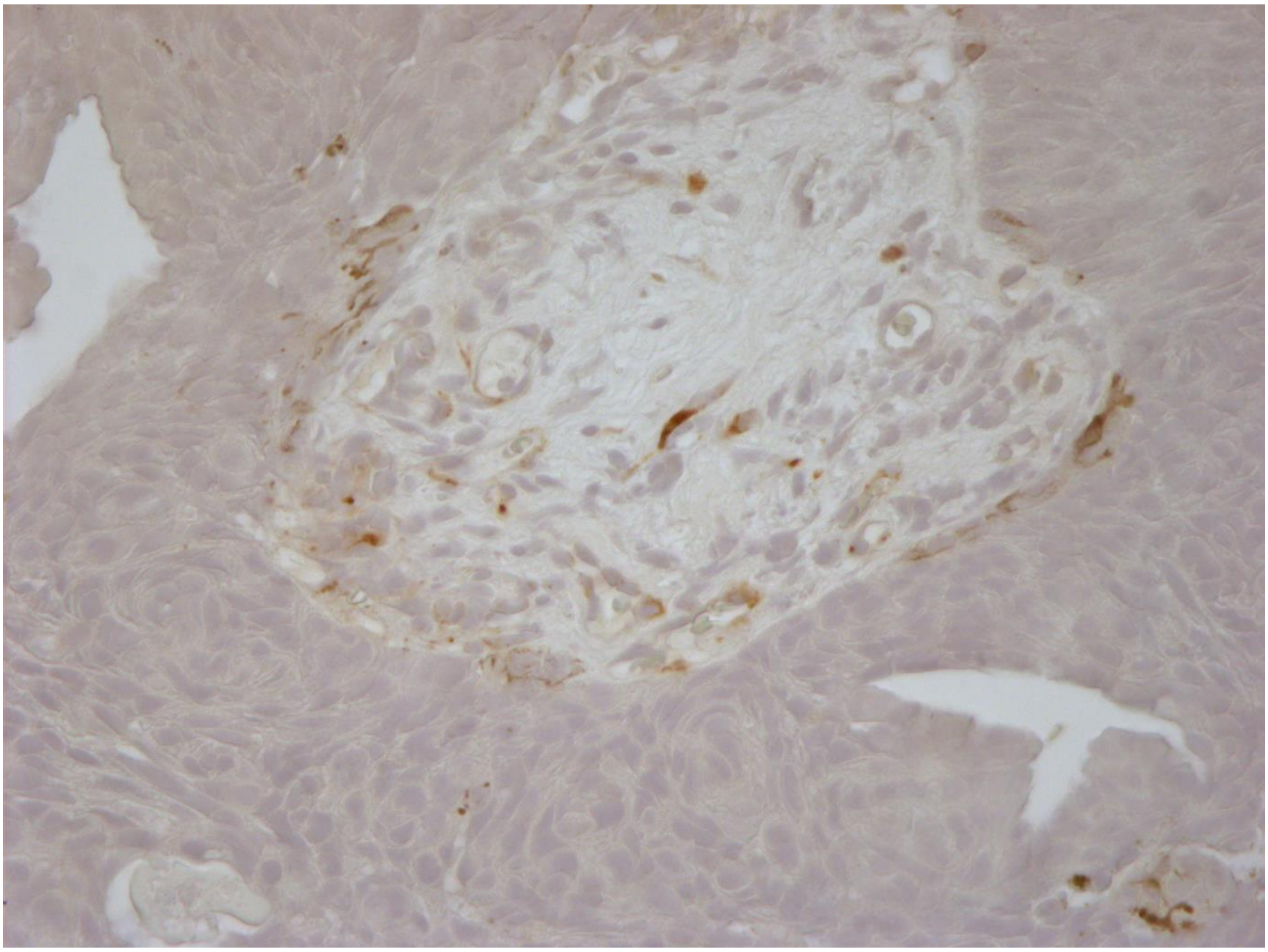

**Figure S3.** Immunofluorescence for CD44 antibody shows spreading of neoplastic cells in the stroma. Some mesenchymal cells are CD44- positive too (original magnification ×200).

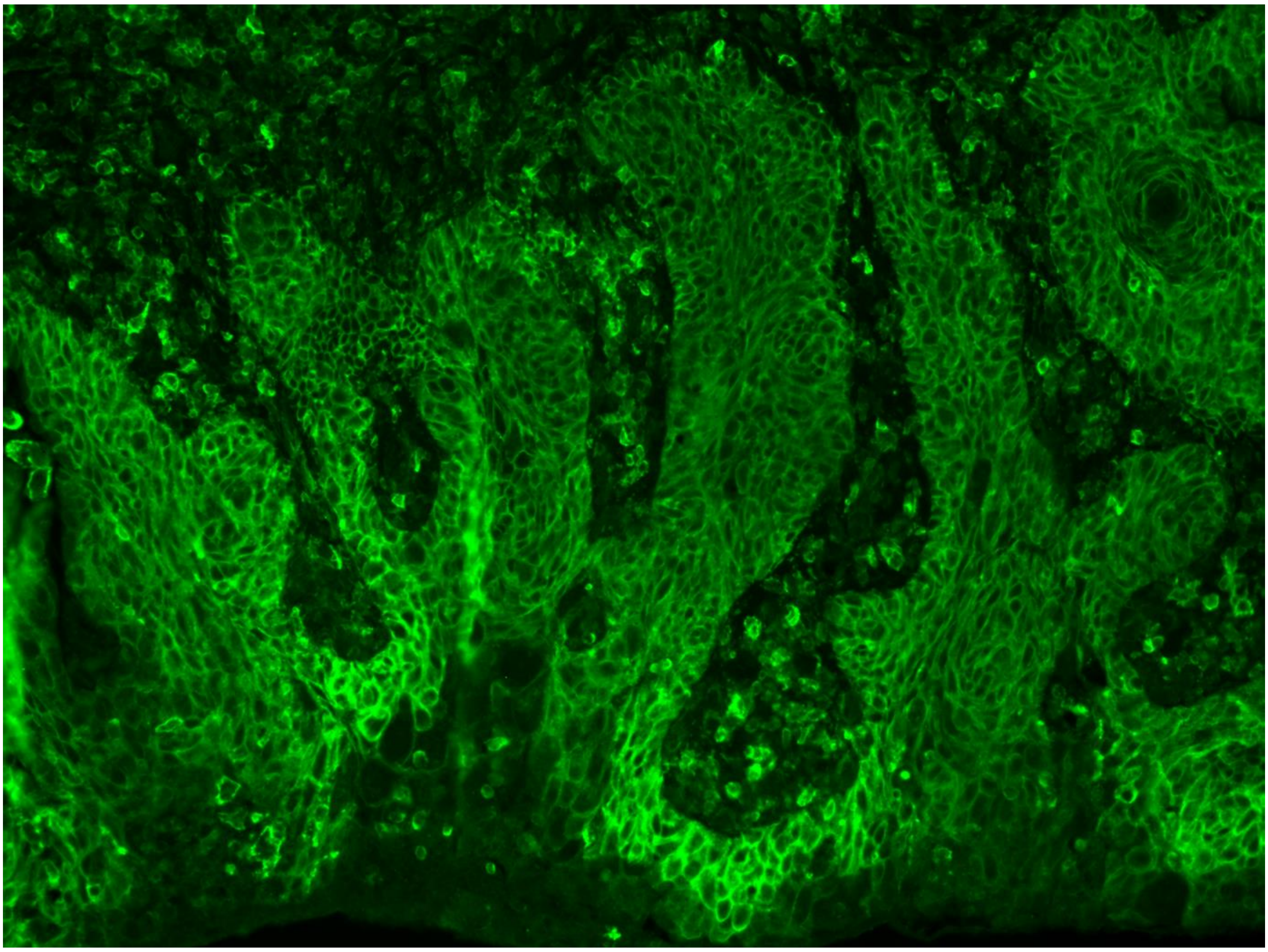

Supplement: Supplementary file 1 — EMT represents a general physiopathologic event implicated in all types of carcinoma and provides them an additional survival advantage. By means of EMT the epithelial tumor cells can transdifferentiate into myofibroblasts, producing the desmoplastic stroma which is essential for tumor growth, invasion and metastasis. During the EMT process, cancer epithelial cells acquire stem cell-like traits and appear positive for putative cancer stem cell (CSC) markers, resulting in a migratory cell phenotype. So EMT- like properties of carcinoma, showed by c- kit staining, are more persuasive when they are associated with an increase of putative stemness marker expression. For this reason we have analyzed nestin, CD44 and CD133 antigens. [file 453874.f1.pdf]
